# Perceived Impact as the Underpinning Mechanism of the End-Spurt and U-Shape Pacing Patterns

**DOI:** 10.3389/fpsyg.2019.01082

**Published:** 2019-05-08

**Authors:** Aviv Emanuel

**Affiliations:** School of Psychological Sciences, Tel Aviv University, Tel Aviv, Israel

**Keywords:** motivation, pacing, end-spurt, U-shape, effort

## Abstract

The end-spurt and U-shape reflect common pacing patterns across a variety of fields (e.g., running, swimming, cycling). To date, however, the literature lacks a clear, parsimonious account for these effects. Here, I propose these pacing patterns can be accounted for by a psychological mechanism termed perceived impact. As athletes perceive their actions to better affect task-progress, they become more motivated and perform better accordingly. To illustrate, if an athlete has five more laps to go during a race, completing the current lap closes 20% of the remaining distance. Alternatively, when she has two more laps to go, the current lap closes 50% of the remaining distance. In the latter case, the impact of completing a single lap on task-progress is perceived to be higher. Her motivation will increase accordingly near the end of the track – giving rise to an end-spurt. I demonstrate the mechanism’s predictive power by reproducing previous findings through simulations. I then move to discuss how this framework is theoretically insightful in view of previous accounts such as the Central Governor Model and the Psyco-Biological Model. I conclude this work with applied strategies for practitioners in their daily routines.

## Introduction

People set goals in their everyday lives. For example, a researcher might set the goal of submitting a grant proposal before a deadline. If she got only one week left, she will likely work hard writing the grant despite severe sleep deprivation. Athletes also set goals in competetive events. For example, in a one-mile race, an athlete might set the goal of “finish the race as fast as possible.” If she got only one lap to go, she will likely perform at her best despite extreme pain. The latter example reflects a pacing pattern known as an “end-spurt” (e.g., Tucker et al., 2006). End-spurts are commonly preceded by an initial decline in performance, from the starting point of a task to its middle; a pacing pattern known as a “U-shape” (e.g., McGibbon et al., 2018).

I suggest the end-spurt and the related U-shape patterns in pacing stem from a simple motivational mechanism – perceived impact: the perceived extent by which one’s actions influence goal-progress. This mechanism is demonstrated in everyday lives and in competitive sports (e.g., when consumers walk toward the end of an aisle in a supermarket; Van Den Bergh et al., 2016; Open-water swimmers during the world championship; Veiga et al., 2019). Indeed, exercise scientists previously suggested that the end-spurt mostly stems from psychological rather than physiological mechanisms (e.g., centrally induced fatigue, perception of effort, concious experience; Noakes, 2012; Smirmaul et al., 2013; Renfree et al., 2014).

Notably, coaches rarely rely on scientific work in their practice (e.g., Stoszkowski and Collins, 2016); which led to suggestions such as including case studies in quantitative research (Halperin, 2018). Some researchers further emphasized that single-case designs could be usefull in applied settings to identify applied principles, orient practice and develop applied intervention procedures for both team and individual sports (e.g., Di Fronso et al., 2016). One reason for this behavior might be the complexity of scientific theories. Previous models of pacing behavior are rather complex – consisting of numerous factors (e.g., rate of heat accumulation, level of motivation, extent of fluid loss, degree of self-belief, extent of muscle fatigue, emotional state, extent of mental fatigue, caffeine intake, amphetamines intake, concious/non-concious experiences, etc., Noakes, 2012; Edwards and Polman, 2013) – and hence are harder to interpret and apply in the field. Due to its simplicity, the proposed mechanism of perceived impact helps closing the gap between coaches and researchers – it consists of only one, easily interpretable factor, and can be efficiently implemented in practice.

In the present work, I first discuss the end-spurt and U-shape patterns in sports science and psychology. I then move to discuss the theoretical framework of the perceived impact mechanism and exemplify how it can replicate findings from Tucker et al. (2006) using simulations. I discuss the mechanism in view of models from exercise and sports science such as the Psycho-Biological Model (PBM; Smirmaul et al., 2013), the Central Governor Model (CGM; Noakes, 2012) and conciousness based accounts (e.g., Edwards and Polman, 2013). Last, I discuss how the present mechanism integrates, differentiates and extends these accounts and suggest practical implications to the field.

## The End-Spurt and U-Shape: an Overview

The end-spurt refers to athletes’ tendency to increase effort (i.e., velocity, power output) closer to the end-point of a physical task (Noakes, 2012; Edwards and Polman, 2013). An end-spurt is commonly preceded by an initial decline in effort from the starting point to the middle of the task; which gives rise to a U-shape pattern. In other words, a U-shape pattern refers to athletes’ tendency to decrease effort mid-way through a physical task, yet increase their effort back again near its end (Edwards and Polman, 2013). The end-spurt pattern in pacing was reliably documented in a variety of domains: repetitive maximal voluntary contractions (Halperin et al., 2014), repetitive self-paced concentric knee extension (Froyd et al., 2016), first serve velocity in tennis (Martin et al., 2019), races of 1600 m and up to 10 k (Tucker et al., 2006), rowing of 2000 m (Garland, 2005; Muehlbauer et al., 2010), swimming of 400 m and up to 25 k (McGibbon et al., 2018; Veiga et al., 2019) and cycling (Foster et al., 2004). For example, a typical 1600 m race athlete would start at a high velocity in the first lap, decrease his velocity during the second and third lap, but would increase his velocity again in the final lap (demonstrating an end-spurt which is a part of a U-shape pattern; Tucker et al., 2006).

The end-spurt, as well as the U-shape patterns, were also studied in psychology. This literature, however, remained mostly uncited in sports science. A large body of psychological research referred to end-spurts as “goal gradients” (Hull, 1932, 1934, 1938); which are defined as the tendency of both animals and humans to increase their effort near their goal’s end state (e.g., Brown, 1948; Losco and Epstein, 1977; Bonezzi et al., 2011; for a review see Heilizer, 1977). For example, when presented with food, mice increase their running velocity (e.g., Hull, 1934) and exert more pulling strength (e.g., Brown, 1948) the closer they are to the track’s end. Similarly, humans increase their walking speed as they approach the end of an aisle in a supermarket (Van Den Bergh et al., 2016); are more likely to donate money to a charity campaign if they are told the target amount is about to be reached (Koo and Fishbach, 2008; Cryder et al., 2013); and perform better in solving simple math problems near the end of their task (Catalano, 1974). People also buy coffee more frequently near to the end of their stamp-card (Kivetz et al., 2006; see Koo and Fishbach, 2012, for similar results with sushi).

A number of studies also referred to U-shape patterns in effort allocation (e.g., Muehlbauer et al., 2010; McGibbon et al., 2018) as the “stuck-in-the-middle” effect. For example, students perform better in a “words in a word” game; successfully constructing more words out of the target word in the beginning and end of a nine-word series, compared to its middle (Bonezzi et al., 2011; Study 1). People were also found to demonstrate a stuck-in-the-middle pattern when correcting typos through a series of nine articles; finding typos at a higher rate in the beginning and end of the series compared to its middle (Bonezzi et al., 2011; Study 3). The stuck-in-the-middle effect was also found while following religious traditions: Jewish students were more likely to light Hanukah candles (a Jewish holiday that lasts eight days, in which people traditionally light candles every day) in the first and last days of the holiday compared to its middle (Touré-Tillery and Fishbach, 2012).

Overall, the end-spurt and U-shape patterns in sports reflect specific cases of the goal gradient and stuck-in-the-middle effects in psychology, when the latter are observed in physical tasks. These two patterns, however, were rarely discussed in the same context in the sports science literature, and received little attention regarding their common underlying mechanisms.

## Theoretical Grounds of the Perceived Impact Mechanism

Psychologists suggested that the goal gradient and stuck-in-the-middle patterns stem from a common mechanism: the perceived impact of one’s current action^1^. The perceived impact mechanism relies on phenomena from three domains: motivation, perception, and progress-monitoring.

Expectancy-value models from the motivation literature (e.g., Atkinson, 1957; see Feather, 1982 for a review) suggest that motivation increases with higher influence actions are expected to have on goal progress (e.g., “how plausible it is that I can achieve this goal”/“how hard would it be for me to achieve this goal?”). For example, a 12-year old who is doing well relative to his class in football, is more likely to continue and play football later on in his life. In other words, motivation increases with higher perceived impact (e.g., perceived impact as competence; Ryan and Deci, 2000; perceived impact as self-efficacy; Bandura, 1977).

In goal-pursuits, a unit of progress is referred to as a “step” toward task completion. In the case of a goal gradient, the perceived impact of the current step is determined by the ratio between this step and the required steps for reaching the task’s end (e.g., reading one page out of 200; completing the first lap out of four; Förster et al., 1998; Heath et al., 1999).

As one gets closer to task completion, each step is perceived to reduce a larger portion of the remaining gap to the task’s end. For example, the first step out of five (i.e., five steps to go) reduces 20% of the five-step gap; whereas the fourth step (i.e., two steps to go) reduces 50% of the two-step gap to the task’s end. A step is perceived as more impactful the larger the gap it reduces, and correspondingly induces higher levels of motivation (e.g., Förster et al., 1998; Heath et al., 1999; Koo and Fishbach, 2012; Cryder et al., 2013).

The stuck-in-the-middle effect is explained by the same mechanism on a broader context of progress-monitoring. Generally, people monitor their rate of progress in goal-pursuits (e.g., Carver and Scheier, 2002). In goal gradients, people use the task’s end for progress-monitoring. Accordingly, if the starting point serves as the reference for monitoring progress, then closer to the starting point perceived impact would be higher and hence, motivation would be higher than later on (e.g., reading one page out of two so far; completing one lap out of three so far; Bonezzi et al., 2011; Koo and Fishbach, 2012). If both the starting and end points can serve as references, people tend to use the nearest reference point for progress-monitoring, or in other words, follow “the small area” principle (Koo and Fishbach, 2012). In such cases, people would use the starting point as a reference as they start, and then switch to use the end point as a reference mid-way through the task (Bonezzi et al., 2011). This behavior gives rise to the stuck-in-the-middle effect: actors invest more effort in the beginning of the task and toward its end (when a reference point is close) and less through the task’s middle (where both reference points are far).

## Formulation of the Perceived Impact Mechanism

To illustrate, perceived impact of a step in goal-pursuit can be stated formally by a simple function. For example, let a step in goal-progress be equal to one, *s* be a series of numbers in an increasing order, representing the index of each step (e.g., *s* = 1, 2, 3, 4, 5, 6, 7), and *PI*_*s*_*i*__ a number between 0 to 100, representing the percent of perceived impact of the current step *s*_*i*_ (e.g., *PI*_*s*_*i*__ = 50%; representing the impact of the current step out of the maximum possible impact a step can have on goal-progress). Accordingly, min(*s*) and max(*s*) are the smallest and highest values in *s*, which represent the starting- and ending-points, respectively. According to the small area principle, people use the nearest reference point, and tend to switch between the beginning and ending points in the middle of the task. Therefore, if *s_i_* < $\frac{\max(s)}{2}$, then:

(1) $${\mathrm{PI}}_{s_{\text{i}}}=\frac{1}{\min(s)+s_{\text{i}}}$$

Else:

(2) $${\mathrm{PI}}_{s_{\text{i}}}=\frac{1}{\max(s)-s_{\text{i}}}$$

**FIGURE 1:**
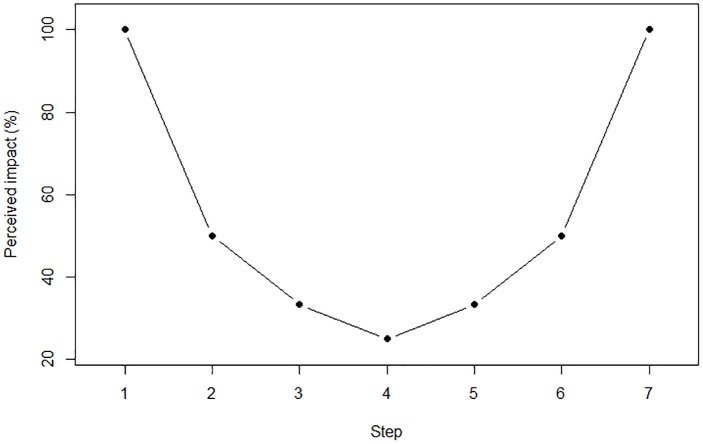
Percent of perceived impact in each step within a seven steps series. In the beginning of the task, the starting point is used as a reference (step 1), thus the percieved impact is higher near the beginning. The reference point is then switched when crossing the middle (step 4) to the end point (step 7) of the task, and hence percieved impact grows as the end point draws near. Overall, this creates a U-shape pattern in percieved impact and therefore a U-shape/stuck-in-the-middle pattern in motivation and effort exertion.

Figure 1 depicts *PI*_*s*_*i*__ through each of a seven steps series according to the formula. As noted earlier, the pattern of perceived impact through goal progress corresponds to U-shape/the stuck-in-the-middle pattern.

This formula can be further adjusted to simulate real-world data. To illustrate, I partially reproduced Figure 5 from Tucker et al. (2006). Tucker et al. (2006) analyzed pacing patterns in men’s world record performances in 5000-m (*n* = 32) and 10,000-m (*n* = 34) events between the years 1921 to 2004^2^. In addition, they presented unpublished data from one mile events (sample size not stated). Figure 5 from Tucker et al.’s (2006) paper presents mean running speed by event interval in each of the three datasets. Their results indicated that the final interval was faster than the preceding one, and the overall pacing pattern corresponded to a U-shape across all intervals in all events from one-mile to 10,000 m.

**FIGURE 2:**
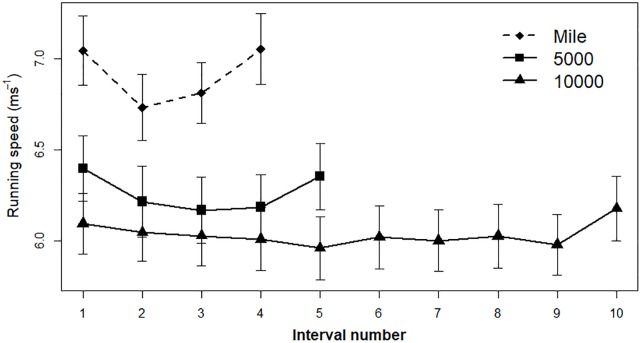
Simulation results based on Tucker et al.’s (2006) Figure 5. Average running speed divided by intervals during men world-record performances in one-mile, 5000-m, and 10,000-m events. Error bars represent 95% confidence interval.

The simulations followed the formula in equations (1) and (2). A step was defined as a progress of 100 m in a race, e.g., a one mile event contained 16 steps. Three additional parameters were added to the model: a constant (*k*) which controls for the average pace in each event, a scaling factor which controls for the steepness of the curve in each event, and a random noise factor which controls for the between-subject variance in each step. The simulation produced three separate datasets for each event (i.e., one-mile, 5000-m, 10,000-m) according to the sample size stated in Tucker et al. (2006), except for the one-mile event in which I estimated the sample size to be 30 (see Figure 2). The R synax used for the simulations and generated datasets are available in the Supplemental Materials section^2^.

In order to determine a U-shape pattern, I tested a quadratic regression for each of the events; a U-shape is indicated by a significant interval^3^ coefficient in the regression model. The regression results indicated a U-shape pattern in all events (see Table 1). Next, I tested for an end-spurt by a paired sample *t*-test that compared the last interval to its preceding one in each event. In all events, the last interval was faster than its preceding interval: one-mile, *t(*29) = −3.45, *p* = 0.001, *d* = 0.62; 5000-m, *t*(31) = −4.41, *p* < 0.001, *d* = 0.78; 10,000-m, *t(*33) = −4.35, *p* < 0.001, *d* = 0.74. These results indicated an end-spurt in all events.

**Table 1:** A summary of the quadratic regression results in each of the simulated events.

Event	Term	*B*	*SE*	*T*	*p*
One-mile	Interval	−0.682	0.110	−6.17	<0.001
	Interval^2^	0.138	0.021	6.37	<0.001
5000-m	Interval	−0.338	0.045	−7.37	<0.001
	Interval^2^	0.054	0.007	7.25	<0.001
10,000-m	Interval	−0.068	0.013	−4.91	<0.001
	Interval^2^	0.006	0.001	5.18	<0.001

Overall, the simulated data that was generated according to the perceived impact model succesfully replicated Tucker et al. (2006) results, demostrating an end-spurt which was a part of a more general U-shape pattern.

## Integration of the Perceived Impact Mechanism With Previous Models

Notably, the end-spurt was previously mentioned in the context of fatigue related models in sports science: (1) the CGM (Noakes and Marino, 2007; Tucker and Noakes, 2009; Noakes, 2012), according to which the brain induces fatigue in the course of a task in order to maintain homeostasis, and (2) the PBM (Marcora, 2008, 2010; Marcora and Staiano, 2010; Smirmaul et al., 2013), according to which the perception of effort increases in the course of a task until reaching the limit of potential motivavtion, which determines task disengagement.

Although differ in their theoretical approaches, both the CGM and the PBM share a number of common principles that may underly the end-spurt phenomena: (1) both may suggest that end-spurts occur due to a reduction of uncertainty regarding the amount of effort to be exerted closer to the end-point (e.g., Smirmaul et al., 2013), and (2) pacing patterns are caused due to a brain based mechanim, rather than the athletes’ physiology (e.g., lack of available muscle energy, an increase in blood lactate, etc., Noakes and Marino, 2007; Tucker and Noakes, 2009; Noakes, 2012).

The perceived impact mechanism does not contradict either one of the CGM or the PBM frameworks; perceived impact can account for the end-spurt and U-shape patterns in view of both. According to the CGM, for example, effort can be regarded as more effective the less its investment risks homeostasis. Hence, as the end of a race draws near, time until task disengagement decreases along with the risk to homeostasis – making effort perceived as more impactful. According to the PBM, for example, investing effort is perceived to be more effective closer to the end of a race because it is less likely for an athlete to reach the point of exhaustion before the race ends (i.e., for the perception of effort to increase past the limit set by potential motivation). Of note, the perceived impact mechanism is theoretically distinct; and emphasizes motivational rather than affective changes in the course of a task, as underlying pacing patterns (e.g., PBM – perception of effort, CGM – fatigue).

Other mechanisms were also suggested to account for the end-spurt, some partially rely on previous models such as the CGM. These accounts emphasize conciousness and decision-making as the determinants of pacing behavior (e.g., Edwards and Polman, 2013; Renfree et al., 2014; Micklewright et al., 2017). For example, the decision to increase effort near the end of the race is determined by the athlete’s decision, made through different levels of consciousness. This decision may itself be the result of a heuristic – a rule of thumb in decision making used instead of deliberate thinking (e.g., “one must increase effort near the end of the race”). These accounts explicitly state possible mechanisms underlying the end-spurt. However, as was previously noted, mice were also found to demonstrate an end-spurt. This suggests that: (1) a relatively low level of consciousness is involved in this effect, and (2) heuristics-based models may not be sufficient to account for this behavior, as they were studied and applied specifically in humans.

In general, consciousness related models are of high theoretical value, yet offer a complex and maybe even an untestable account for the end-spurt. It is unclear how the consciousness factor would affect pacing behavior (e.g., would a decision made in low rather than high consciousness levels increase the magnitude of the end-spurt?). The perceived impact mechanism accounts for the end-spurt parsimoniously using a single factor (i.e., an increase in motivation with proximity to a reference point), which was validated in the literature using experimental data, and, as demonstrated above, is able to conceptually replicate the results of former studies through simulations^4^.

## Practical Implication of the Perceived Impact Mechanism

The perceived impact mechanism offers strong, field-relevant predictions regarding athletes’ level of motivation through physical tasks. It can therefore offer practical applications for both coaches and athletes. For example, an athletes is expected to maximize her performance near the starting-point of a race, when instructed to monitor her progress in reference to the distance passed so far (e.g., “one lap completed”). Similarly, she is also expected to maximize performance when instructed to monitor progress in reference to the remaining distance to the end of the race near the end point (e.g., “one lap to go”). This prediction is partially supported by previous findings in the field of consumer behavior (e.g., Koo and Fishbach, 2012).

Apart from athletes, the mechanism also offers relevant implications for the general population. People naturally tend to adopt arbitrary reference points when performing a demanding physical task (e.g., Allen et al., 2016). Trainers can utilize this tendency to increase their trainees’ motivation and performance in training sessions, by simply counting the set repetitions or seconds in either an ascending or a descending order. For example, when assigning a trainee with the task of doing 20 squats, a trainer can count the repetitions in an ascending order from repetition one to 10 (e.g., “one,” “two,” “three”), and then switch to count in a descending order (e.g., “three,” “two,” “one”). On a broader perspective, trainees might tend to be more motivated in the beginning and end of a training session. Trainers can thereby construct the whole training session accordingly – with challenging exercises assigned to the beginning and end of the session, while easier exercises assigned to its middle.

## Conclusion

This work reviewed the end-spurt and U-shape patterns both in the physical and mental domains, and suggests a unifying framework for their understanding – the perceived impact mechanism. This account builds upon a single motivational factor, perceived impact, as the main determinant of these patterns both in sports science and psychology, and bears high theoretical and practical value.

## Author Contributions

AE wrote the manuscript, simulations and analyzed the data.

## Conflict of Interest Statement

The author declares that the research was conducted in the absence of any commercial or financial relationships that could be construed as a potential conflict of interest.

## Footnotes

1 I will refer perceived impact of one’s current action as perceived impact.

2 The authors also analyzed the pacing pattern of an 800-m event in which end-spurts were not found. Hence, these events were not included in the model.

3 The simulation includes an option to adjust parameters given real data.

4 Of note, the mentioned phenomena are discussed in the context of human physiological constraints, and the mechanism accounts only for situations in which an increase in effort near the end of a task was observed so far (e.g., 10 k races).
