# Corrigendum: Perceived Impact as the Underpinning Mechanism of the End-Spurt and U-Shape Pacing Patterns

**DOI:** 10.3389/fpsyg.2019.01597

**Published:** 2019-07-09

**Authors:** Aviv Emanuel

**Affiliations:** School of Psychological Sciences, Tel Aviv University, Tel Aviv, Israel

**Keywords:** motivation, pacing, end-spurt, U-shape, effort

In the original article, there was an error. In equations (1) and (2), the nominator should have been “1.” In addition, the text should have clearly indicated this fact.

A correction has been made to the **Formulation of the Perceived Impact Mechanism** section, paragraph one:

“To illustrate, perceived impact of a step in goal-pursuit can be stated formally by a simple function. For example, let a step in goal-progress be equal to one, *s* be a series of numbers in an increasing order, representing the index of each step (e.g., *s* = 1, 2, 3, 4, 5, 6, 7), and *PI*_*s*_*i*__ a number between 0 to 100, representing the percent of perceived impact of the current step *s*_*i*_ (e.g., *PI*_*s*_*i*__ = 50%; representing the impact of the current step out of the maximum possible impact a step can have on goal-progress). Accordingly, min(*s*) and max(*s*) are the smallest and highest values in *s*, which represent the starting- and ending-points, respectively. According to the small area principle, people use the nearest reference point, and tend to switch between the beginning and ending points in the middle of the task. Therefore, if $s_{i}<\frac{\max\left(s\right)}{2}$, then:

(1) $$\begin{array}{r} PI_{s_{i}}=\frac{1}{\min\left(s\right)+s_{i}} \end{array}$$

Else:

(2) $$\begin{array}{r} PI_{s_{i}}=\frac{1}{\text{max }\left(s\right)\;-\;s_{i}} \end{array}$$

The author apologizes for this error and state that this does not change the scientific conclusions of the article in any way. The original article has been updated.

