# Supplementary material for: Perceived Impact as the Underpinning Mechanism of the End-Spurt and U-Shape Pacing Patterns
Source: Front Psychol. 2019 May 8;10:1082. doi: 10.3389/fpsyg.2019.01082 (PMC6519309; doi:10.3389/fpsyg.2019.01082)
Supplement: Supplementary file 1 [file Table_1.DOCX]

library(dplyr)

library(tidyr)

library(ggplot2)

library(lme4)

library(Hmisc)

library(lsr)

library(stringr)

library(apaTables)

#A partial simulation of the Figure 5 from Tucker et al. (2006)

set.seed(1)

####one mile####

number_of_sims <- 1

segments <- seq(from = 0, to = 16, by = 4)

results_1600 <- matrix(NA, nrow = number_of_sims, ncol = length(segments))

total_steps <- 1:16

n <- 30 #note: sample size is not specified in Tucker et al. (2006)

k <- 6.7 # constant adjusting for overall mean pace. For example, a mean pace in 800-meters race is faster than that of a 10k race.

scale_factor <- 1.7 #a variable that adjusts the steepness of the U-shape

perceived_impact <- matrix(NA, nrow = n, ncol = length(total_steps))

for (s in 1:number_of_sims){

for (j in 1:n){

for (i in total_steps) {

# for (j in n){

if (i < max(total_steps)/2){

#perceived_impact

perceived_impact[j,i] = (1/((min(total_steps)+i)-1)/scale_factor)+k+rnorm(1, mean = 0, sd = .5)

}

else {

perceived_impact[j,i] = (1/((max(total_steps)-i)+1)/scale_factor)+k+rnorm(1, mean = 0, sd = .5)

}

}}

for (p in total_steps) {

mean_PI[p] <- mean(perceived_impact[,p])

}

segments_mean <- rep(NA, 4)

#segments<-c(0,10)

for (m in 1:(length(segments)-1)){

results_1600[s,m] <- mean(mean_PI[(segments[m]+1):segments[m+1]])

}

}

####plot across multiple simulations####

one_mile_df <- data.frame("sim_id" = 1:number_of_sims, "interval1" = results_1600[,1], "interval2" = results_1600[,2], "interval3" = results_1600[,3], "interval4" = results_1600[,4] )

#if we choose to run more than one simulation:

segments_mean <- as.numeric(colMeans(one_mile_df[,2:5]))

se_segment <- as.numeric(apply(one_mile_df[,2:5], 2, sd))

se_segment <- se_segment/sqrt(number_of_sims)

intervals <- 1:4

#plot(intervals, segments_mean,type = "b", col="black" ,xlab="Interval number",pch= 19 ,bg = "black",ylab= expression(paste("Running speed (ms"^-1,")")))

errbar(intervals, segments_mean,segments_mean+se_segment,segments_mean-se_segment,type = "b", col="black" ,xaxt = 'n',xlab="Interval number",pch= 19 ,bg = "black",ylab= expression(paste("Running speed (ms"^-1,")")))

axis(1, at=1:4,labels=c("1", "2", "3", "4"))

lines(intervals, segments_mean,type = "b", col="black", add = T) # adds a line for health expenditures

####plot for a single simulation####

segments_mean_1_6 <- rep(NA, 4)

#segments<-c(0,10)

for (i in 1:(length(segments)-1)){

segments_mean_1_6[i] <- mean(mean_PI[(segments[i]+1):segments[i+1]])

}

segments_mean_1_6

se_segment_1_6 <- rep(NA, 4)

for (i in 1:(length(segments)-1)) {

se_segment_1_6[i] <- sd(perceived_impact[,(segments[i]+1):segments[i+1]])/sqrt(n)

}

se_segment_1_6

#xlim = c(0.7,7.3), ylim = c(22,103))

intervals_1_6 <- 1:4

#plot(intervals, segments_mean,type = "b", col="black" ,xlab="Interval number",pch= 19 ,bg = "black",ylab= expression(paste("Running speed (ms"^-1,")")))

errbar(intervals_1_6, segments_mean_1_6,segments_mean_1_6+se_segment_1_6,segments_mean_1_6-se_segment_1_6, cex = 1.7,col="black" ,xaxt = 'n',xlab="Interval number",pch= 18 ,bg = "black",ylab= expression(paste("Running speed (ms"^-1,")")))

axis(1, at=1:4,labels=c("1", "2", "3", "4"))

lines(intervals, segments_mean,lty =2,lwd = 2, col="black", add = T) # adds a line for health expenditures

####statistical analysis####

#create a data frame for analysis

interval_speed <- matrix(NA, ncol = length(intervals), nrow = n)

for (j in 1:n){

for (i in 1:(length(segments)-1)){

interval_speed[j,i] <- mean(perceived_impact[j,(segments[i]+1):segments[i+1]])

}}

interval_speed

one_mile_df_analsyis <- data.frame("sim_id" = 1:n, "interval1" = interval_speed[,1], "interval2" = interval_speed[,2], "interval3" = interval_speed[,3], "interval4" = interval_speed[,4] )

t.test(one_mile_df_analsyis$interval3,one_mile_df_analsyis$interval4,paired = T )

cohensD(one_mile_df_analsyis$interval3,one_mile_df_analsyis$interval4, method = "paired")

one_mile_df_regression <- gather(one_mile_df_analsyis, interval, running_speed, -c(sim_id))

one_mile_df_regression$interval <- as.numeric(str_replace(one_mile_df_regression$interval,"interval", ""))

quad_model <- lm(running_speed~ interval + I(interval^2), data =one_mile_df_regression)

summary(quad_model)

mean_PI <- rep(NA, 4)

perceived_impact

for (i in total_steps) {

mean_PI[i] <- mean(perceived_impact[,i])

}

se <- rep(NA, 4)

for (i in total_steps) {

se[i] <- sd(perceived_impact[,i])/sqrt(n)

}

#xlim = c(0.7,7.3), ylim = c(22,103))

plot(total_steps, mean_PI,type = "b", col="black" ,xlab="Interval number",pch= 19 ,bg = "black",ylab= expression(paste("Running speed (ms"^-1,")")))

errbar(total_steps, mean_PI,mean_PI+se,mean_PI-se,type = "b", col="black" ,xaxt = 'n',xlab="Interval number",pch= 19 ,bg = "black",ylab= expression(paste("Running speed (ms"^-1,")")))

axis(1, at=1:4,labels=c("1", "2", "3", "4"))

lines(total_steps, mean_PI,type = "b", col="black", add = T) # adds a line for health expenditures

errbar(time, mean_twelve,mean_twelve+se_twelve,mean_twelve-se_twelve,type = "b", col="black" ,ylim = c(min(c(mean_twelve-se_twelve,mean_six - se_six)), max(c(mean_twelve+se_twelve,mean_six + se_six))),xlab="Segment",pch= 19 ,bg = "black",xaxt = 'n',ylab="Standardized key presses per segment",xlim = c(0.7,7.3)) # adds titles to the axes

####5000k####

number_of_sims <- 1

segments <- seq(from = 0, to = 50, by = 10)

results_5k <- matrix(NA, nrow = number_of_sims, ncol = length(segments))

total_steps <- 1:50

n <- 32

k <- 6.2 # constant adjusting for overall pace. For example, a pace in 800-meters race is faster than that of a 10k race.

scale_factor <-2 #a variable that adjusts the steepness of the U-shape

perceived_impact <- matrix(NA, nrow = n, ncol = length(total_steps))

for (s in 1:number_of_sims){

for (j in 1:n){

for (i in total_steps) {

# for (j in n){

if (i < max(total_steps)/2){

#perceived_impact

perceived_impact[j,i] = (1/((min(total_steps)+i)-1)/scale_factor)+k+rnorm(1, mean = 0, sd = .5)

}

else {

perceived_impact[j,i] = (1/((max(total_steps)-i)+1)/scale_factor)+k+rnorm(1, mean = 0, sd = .5)

}

}}

for (p in total_steps) {

mean_PI[p] <- mean(perceived_impact[,p])

}

segments_mean <- rep(NA, 4)

#segments<-c(0,10)

for (m in 1:(length(segments)-1)){

results_5k[s,m] <- mean(mean_PI[(segments[m]+1):segments[m+1]])

}

}

####plot across multiple simulations####

results_5k <-as.data.frame(results_5k)

colnames(results_5k) <- c("interval1","interval2","interval3","interval4","interval5","m")

library(dplyr)

results_5k <- results_5k %>% select(-m)%>% mutate(sim_id = 1:number_of_sims)

#if we choose to run more than one simulation:

segments_mean <- as.numeric(colMeans(results_5k[,1:5]))

se_segment <- as.numeric(apply(results_5k[,1:5], 2, sd))

se_segment <- se_segment/sqrt(number_of_sims)

intervals <- 1:5

#plot(intervals, segments_mean,type = "b", col="black" ,xlab="Interval number",pch= 19 ,bg = "black",ylab= expression(paste("Running speed (ms"^-1,")")))

errbar(intervals, segments_mean,segments_mean+se_segment,segments_mean-se_segment,type = "b", col="black" ,xaxt = 'n',xlab="Interval number",pch= 19 ,bg = "black",ylab= expression(paste("Running speed (ms"^-1,")")))

axis(1, at=1:5,labels=c("1", "2", "3", "4","5"))

lines(intervals, segments_mean,type = "b", col="black", add = T) # adds a line for health expenditures

####plot for a single simulation####

segments_mean_5 <- rep(NA, 5)

for (i in 1:(length(segments)-1)){

segments_mean_5[i] <- mean(mean_PI[(segments[i]+1):segments[i+1]])

}

segments_mean_5

se_segment_5 <- rep(NA, 5)

for (i in 1:(length(segments)-1)) {

se_segment_5[i] <- sd(perceived_impact[,(segments[i]+1):segments[i+1]])/sqrt(n)

}

se_segment_5

intervals <- 1:5

errbar(intervals, se_segment_5,se_segment_5+se_segment_5,se_segment_5-se_segment_5, cex = 1.7,col="black" ,xaxt = 'n',xlab="Interval number",pch= 15 ,bg = "black",ylab= expression(paste("Running speed (ms"^-1,")")))

axis(1, at=1:5,labels=c("1", "2", "3", "4","5"))

lines(intervals, segments_mean,lty =1,lwd = 2, col="black", add = T) # adds a line for health expenditures

####statistical analysis####

#create a data frame for analysis

interval_speed_5 <- matrix(NA, ncol = length(intervals), nrow = n)

for (j in 1:n){

for (i in 1:(length(segments)-1)){

interval_speed_5[j,i] <- mean(perceived_impact[j,(segments[i]+1):segments[i+1]])

}}

interval_speed_5

interval_speed_5 <-as.data.frame(interval_speed_5)

colnames(interval_speed_5) <- c("interval1","interval2","interval3","interval4","interval5")

library(dplyr)

interval_speed_5 <- interval_speed_5 %>% mutate(sim_id = 1:n)

t.test(interval_speed_5$interval4,interval_speed_5$interval5,paired = T )

cohensD(interval_speed_5$interval4,interval_speed_5$interval5, method = "paired")

five_mile_df_regression <- gather(interval_speed_5, interval, running_speed, -c(sim_id))

five_mile_df_regression$interval <- as.numeric(str_replace(five_mile_df_regression$interval,"interval", ""))

quad_model <- lm(running_speed~ interval + I(interval^2), data =five_mile_df_regression)

summary(quad_model)

####10000k####

number_of_sims <- 1

segments <- seq(from = 0, to = 100, by = 10)

results_10k <- matrix(NA, nrow = number_of_sims, ncol = length(segments))

total_steps <- 1:100

n = 34

k<- 6

scale_factor <- 2 #a variable that adjusts the steepness of the U-shape

#rep(NA,max(total_steps)

perceived_impact <- matrix(NA, nrow = n, ncol = length(total_steps))

for (s in 1:number_of_sims){

for (j in 1:n){

for (i in total_steps) {

# for (j in n){

if (i < max(total_steps)/2){

#perceived_impact

perceived_impact[j,i] = (1/((min(total_steps)+i)-1)/scale_factor)+k+rnorm(1, mean = 0, sd = .5)

}

else {

perceived_impact[j,i] = (1/((max(total_steps)-i)+1)/scale_factor)+k+rnorm(1, mean = 0, sd = .5)

}

}}

for (p in total_steps) {

mean_PI[p] <- mean(perceived_impact[,p])

}

segments_mean <- rep(NA, 10)

#segments<-c(0,10)

for (m in 1:(length(segments)-1)){

results_10k[s,m] <- mean(mean_PI[(segments[m]+1):segments[m+1]])

}

}

####plot across multiple simulations####

#fivek_df <- data.frame("sim_id" = 1:number_of_sims, "interval1" = results_1600[,1], "interval2" = results_1600[,2], "interval3" = results_1600[,3], "interval4" = results_1600[,4] )

results_10k <-as.data.frame(results_10k)

colnames(results_10k) <- c("interval1","interval2","interval3","interval4","interval5","interval6","interval7","interval8","interval9","interval10","m")

library(dplyr)

results_10k <- results_10k %>% select(-m)%>% mutate(sim_id = 1:number_of_sims)

#if we choose to run more than one simulation:

segments_mean <- as.numeric(colMeans(results_10k[,1:10]))

se_segment <- as.numeric(apply(results_10k[,1:10], 2, sd))

se_segment <- se_segment/sqrt(number_of_sims)

intervals <- 1:10

#plot(intervals, segments_mean,type = "b", col="black" ,xlab="Interval number",pch= 19 ,bg = "black",ylab= expression(paste("Running speed (ms"^-1,")")))

errbar(intervals, segments_mean,segments_mean+se_segment,segments_mean-se_segment,type = "b", col="black" ,xaxt = 'n',xlab="Interval number",pch= 19 ,bg = "black",ylab= expression(paste("Running speed (ms"^-1,")")))

axis(1, at=1:10,labels=c("1", "2", "3", "4","5","6","7","8","9","10"))

lines(intervals, segments_mean,type = "b", col="black", add = T) # adds a line for health expenditures

####plot for a single simulation####

segments_mean_10 <- rep(NA, 10)

#segments<-c(0,10)

for (i in 1:(length(segments)-1)){

segments_mean_10[i] <- mean(mean_PI[(segments[i]+1):segments[i+1]])

}

segments_mean_10

se_segment_10 <- rep(NA, 10)

for (i in 1:(length(segments)-1)) {

se_segment_10[i] <- sd(perceived_impact[,(segments[i]+1):segments[i+1]])/sqrt(n)

}

se_segment_10

#xlim = c(0.7,7.3), ylim = c(22,103))

intervals_10 <- 1:10

#plot(intervals, segments_mean,type = "b", col="black" ,xlab="Interval number",pch= 19 ,bg = "black",ylab= expression(paste("Running speed (ms"^-1,")")))

errbar(intervals, segments_mean,segments_mean+se_segment,segments_mean-se_segment, cex = 1.7,col="black" ,xaxt = 'n',xlab="Interval number",pch= 25 ,bg = "black",ylab= expression(paste("Running speed (ms"^-1,")")))

axis(1, at=1:10,labels=c("1", "2", "3", "4","5","6","7","8","9","10"))

lines(intervals, segments_mean,lty =1,lwd = 2, col="black", add = T) # adds a line for health expenditures

####statistical analysis####

#create a data frame for analysis

interval_speed_10 <- matrix(NA, ncol = length(intervals), nrow = n)

for (j in 1:n){

for (i in 1:(length(segments)-1)){

interval_speed_10[j,i] <- mean(perceived_impact[j,(segments[i]+1):segments[i+1]])

}}

interval_speed_10

interval_speed_10 <-as.data.frame(interval_speed_10)

colnames(interval_speed_10) <- c("interval1","interval2","interval3","interval4","interval5","interval6","interval7","interval8","interval9","interval10")

library(dplyr)

interval_speed_10 <- interval_speed_10 %>% mutate(sim_id = 1:n)

t.test(interval_speed_10$interval9,interval_speed_10$interval10,paired = T )

cohensD(interval_speed_10$interval9,interval_speed_10$interval10, method = "paired")

tenk_df_regression <- gather(interval_speed_10, interval, running_speed, -c(sim_id))

tenk_df_regression$interval <- as.numeric(str_replace(tenk_df_regression$interval,"interval", ""))

quad_model <- lm(running_speed~ interval + I(interval^2), data =tenk_df_regression)

summary(quad_model)

####everything in one plot! ####

#transforming SEs to CIs

se_segment_10<- se_segment_10*1.96

se_segment_5<- se_segment_5*1.96

se_segment_1_6<- se_segment_1_6*1.96

#ylab= expression(paste("Running speed (ms"^-1,")")),

errbar(intervals_10, segments_mean_10,segments_mean_10+se_segment_10,segments_mean_10-se_segment_10,ylab = '' ,cex = 1.7,col="black" ,xaxt = 'n',xlab="",pch= 17 ,bg = "black", ylim = c(min(segments_mean_10-se_segment_10), max(segments_mean_1_6+se_segment_1_6)),font.lab = 1)

axis(1, at=1:10,labels=c("1", "2", "3", "4","5","6","7","8","9","10"))

lines(intervals_10, segments_mean_10,lty =1,lwd = 2, col="black", add = T) # adds a line for health expenditures

intervals_5 <- 1:5

errbar(intervals_5, segments_mean_5,segments_mean_5+se_segment_5,segments_mean_5-se_segment_5, cex = 1.7,col="black" ,xaxt = 'n',xlab="",pch= 15 ,bg = "black", add = T)

lines(intervals_5, segments_mean_5,lty =1,lwd = 2, col="black", add = T) # adds a line for health expenditures

intervals_1_6 <- 1:4

#plot(intervals, segments_mean,type = "b", col="black" ,xlab="Interval number",pch= 19 ,bg = "black",ylab= expression(paste("Running speed (ms"^-1,")")))

errbar(intervals_1_6, segments_mean_1_6,segments_mean_1_6+se_segment_1_6,segments_mean_1_6-se_segment_1_6, cex = 1.7,col="black" ,xaxt = 'n',xlab="",pch= 18 ,bg = "black",add = T)

lines(intervals_1_6, segments_mean_1_6,lty =2,lwd = 2, col="black", add = T) # adds a line for health expenditures

legend(8,7.25, # places a legend at the appropriate place

c("Mile", "5000","10000"), # gives the legend appropriate symbols (lines)

lwd=c(2.5,2.5,2.5) ,lty = c(2,1,1),pch= c(18,15,17),col=c("black","black","black"),bg = "white",box.lty=0,cex=1.5) # gives the legend lines the correct color and width

mtext(text = expression(paste("Running speed (ms"^-1,")")),

side = 2, #side 2 = left

line = 2.5,font = 2, cex = 1.2)

mtext(text = "Interval number",

side = 1, #side 2 = left

line = 2.5,font = 2, cex = 1.2)

####all statistical analyses:####

#note: results may vary between each specific run of the simulation, but would replicate in the long term

#10k:

t.test(interval_speed_10$interval9,interval_speed_10$interval10,paired = T )

cohensD(interval_speed_10$interval9,interval_speed_10$interval10, method = "paired")

tenk_df_regression <- gather(interval_speed_10, interval, running_speed, -c(sim_id))

tenk_df_regression$interval <- as.numeric(str_replace(tenk_df_regression$interval,"interval", ""))

quad_model <- lm(running_speed~ interval + I(interval^2), data =tenk_df_regression)

summary(quad_model)

#lm.beta(quad_model, standardized = TRUE)

setwd("C:/Users/User/Dropbox/End-Spurt")

write.csv(tenk_df_regression, "tenk_df_regression.csv")

write.csv(interval_speed_10, "tenk_df_ttest.csv")

#5k:

t.test(interval_speed_5$interval4,interval_speed_5$interval5,paired = T )

cohensD(interval_speed_5$interval4,interval_speed_5$interval5, method = "paired")

fivek_df_regression <- gather(interval_speed_5, interval, running_speed, -c(sim_id))

fivek_df_regression$interval <- as.numeric(str_replace(five_mile_df_regression$interval,"interval", ""))

quad_model <- lm(running_speed~ interval + I(interval^2), data =fivek_df_regression)

summary(quad_model)

write.csv(fivek_df_regression, "fivek_df_regression.csv")

write.csv(interval_speed_5, "fivek_df_ttest.csv")

#1.6k:

t.test(one_mile_df_analsyis$interval3,one_mile_df_analsyis$interval4,paired = T )

cohensD(one_mile_df_analsyis$interval3,one_mile_df_analsyis$interval4, method = "paired")

one_mile_df_regression <- gather(one_mile_df_analsyis, interval, running_speed, -c(sim_id))

one_mile_df_regression$interval <- as.numeric(str_replace(one_mile_df_regression$interval,"interval", ""))

quad_model <- lm(running_speed~ interval + I(interval^2), data =one_mile_df_regression)

summary(quad_model)

write.csv(one_mile_df_regression, "one_mile_df_regression.csv")

write.csv(one_mile_df_analsyis, "one_mile_df_ttest.csv")
